# Reclaiming Our Spirits: Development and Pilot Testing of a Health Promotion Intervention for Indigenous Women Who Have Experienced Intimate Partner Violence

**DOI:** 10.1002/nur.21795

**Published:** 2017-04-21

**Authors:** Colleen Varcoe, Annette J. Browne, Marilyn Ford‐Gilboe, Madeleine Dion Stout, Holly McKenzie, Roberta Price, Victoria Bungay, Victoria Smye, Jane Inyallie, Linda Day, Koushambhi Khan, Angela Heino, Marilyn Merritt‐Gray

**Affiliations:** ^1^ Professor University of British Columbia School of Nursing T201 − 2211 Wesbrook Mall Vancouver, BC V6T 2B5 Canada; ^2^ Professor University of British Columbia School of Nursing Vancouver, BC Canada; ^3^ Professor Western University Arthur Labatt Family School of Nursing London, ON Canada; ^4^ Honorary Professor University of British Columbia School of Nursing Vancouver, BC Canada; ^5^ Graduate Research Assistant University of British Columbia School of Nursing Vancouver, BC Canada; ^6^ Elder Researcher University of British Columbia School of Nursing Vancouver, BC Canada; ^7^ Associate Professor University of British Columbia School of Nursing Vancouver, BC Canada; ^8^ Associate Professor Western University Arthur Labatt Family School of Nursing London, ON Canada; ^9^ Addictions Counselor Central Interior Native Health Society Prince George, BC Canada; ^10^ Executive Director Aboriginal Mother Center Society Vancouver, BC Canada; ^11^ Research Manager University of British Columbia School of Nursing Vancouver, BC Canada; ^12^ Professor University of New Brunswick Faculty of Nursing Fredericton, NB Canada

**Keywords:** nursing interventions, violence, abuse, visitation

## Abstract

Indigenous women are subjected to high rates of multiple forms of violence, including intimate partner violence (IPV), in the context of ongoing colonization and neo‐colonization. Health promotion interventions for women who experience violence have not been tailored specifically for Indigenous women. Reclaiming Our Spirits (ROS) is a health promotion intervention designed for Indigenous women living in an urban context in Canada. In this paper, we describe the development of the intervention, results of a pilot study, and the revised subsequent intervention. Building on a theory‐based health promotion intervention (*i*HEAL) showing promising results in feasibility studies, ROS was developed using a series of related approaches including (a) guidance from Indigenous women with research expertise specific to IPV and Indigenous women's experiences; (b) articulation of an Indigenous lens, including using Cree (one of the largest Indigenous language groups in North America) concepts to identify key aspects; and (c) interviews with Elders (*n* = 10) living in the study setting. Offered over 6–8 months, ROS consists of a Circle, led by an Indigenous Elder, and 1:1 visits with a Registered Nurse, focused on six areas for health promotion derived from previous research. Pilot testing with Indigenous women (*n* = 21) produced signs of improvement in most measures of health from pre‐ to post‐intervention. Women found the pilot intervention acceptable and helpful but also offered valuable suggestions for improvement. A revised intervention, with greater structure within the Circle and nurses with stronger knowledge of Indigenous women's experience and community health, is currently undergoing testing. © 2017 The Authors. *Research in Nursing & Health* Published by Wiley Periodicals, Inc.

Globally, Indigenous^1^ (Royal Commission on Aboriginal Peoples, 1996b; United Nations, 2008) women are subjected to high rates of multiple forms of violence, including intimate partner violence (IPV), in the context of historical and ongoing colonization, neo‐colonization, and economic globalization (Anaya, 2012; Kubik, Bourassa, & Hampton, 2009; Kuokkanen, 2008; Valdez‐Santiago, Híjar, Rojas Martínez, Ávila Burgos, & Arenas Monreal, 2013). In Canada, Indigenous women are subject to higher rates of violence than women in the general population; these higher rates are related to historical and ongoing colonization and racism through multiple mechanisms, primarily socioeconomic vulnerability (Brownridge, 2008; Daoud et al., 2012; Daoud, Smylie, Urquia, Allan, & O'Campo, 2013; Kubik et al., 2009; Pedersen, Malcoe, & Pulkingham, 2013).

Health promotion interventions for women who experience violence are being developed and tested with promising results but have not been developed specifically for Indigenous women. Because they fail to account for the multiple traumas and ongoing racism Indigenous women experience (Herring, Spangaro, Lauw, & McNamara, 2013), mainstream services are often inappropriate or even harmful, deterring health care access, and resulting in misdiagnoses and under‐treatment for Indigenous women (Browne, 2007; Browne, Smye et al., 2011; Denison, Varcoe, & Browne, 2014; Firestone, Smylie, Maracle, Spiller, & O'Campo, 2014).

In this paper, we describe the adaptation, pilot testing, and revision of a health promotion intervention for urban Indigenous women who have experienced IPV. At the end of the study, the intervention being tested in the Aboriginal Women's Intervention (AWI) study was renamed by pilot participants “Reclaiming Our Spirits” (ROS). We conclude by describing the study currently testing the efficacy of the refined intervention.

## Development and Testing Context

This study was conducted in one inner city in Canada that is home to diverse Indigenous women, many who have moved from locations throughout North America. Due to regressive social welfare policies and widening income inequities, this inner city is characterized by high levels of poverty and little affordable housing. Negative media attention has pathologized the area's residents (Liu & Blomley, 2013; Peters & Anderson, 2013; Women's Coalition, 2014). Given the sociopolitical, historical, and economic context of Indigenous people in Canada and this setting, we anticipated that Indigenous women in this community would have experienced multiple forms of interpersonal and structural violence, such as extreme poverty, racism, and historical trauma but intended to capitalize on the strength and resilience of the women and to use culture and tradition as sources of strength and healing.

**Figure 1:**
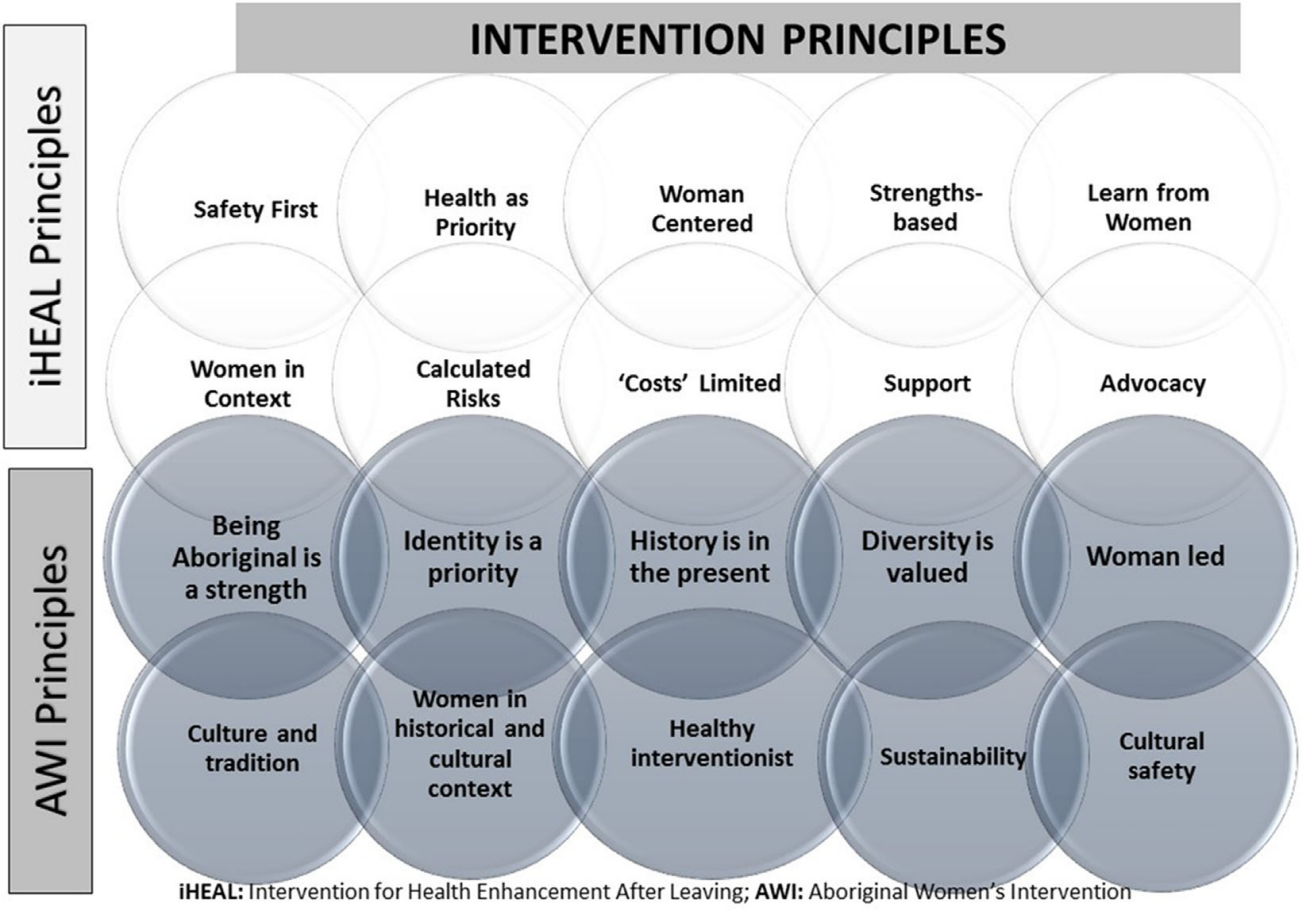
The *i*HEAL and Aboriginal Women's Intervention Study (“Reclaiming Our Spirits”) Principles.

## Building on an Existing Intervention

Previously, our team developed a complex evidence‐ and theory‐based intervention, the Intervention for Health Enhancement After Leaving (*i*HEAL; Ford‐Gilboe, Merritt‐Gray, Varcoe, & Wuest, 2011; Ford‐Gilboe, Wuest, Varcoe, & Merritt‐Gray, 2006; Wuest, Ford‐Gilboe, Merritt‐Gray, & Varcoe, 2013). iHEAL was based on (a) grounded theory of women's health promotion after leaving abusive male partners; (b) our empirical work examining women's health after leaving abusive male partners; and (c) promising health promotion interventions. In our grounded theoretical work, we conceptualized women's health promotion in the aftermath of IPV as a process of Strengthening Capacity to Limit Intrusion (Ford‐Gilboe, Wuest, & Merritt‐Gray, 2005; Wuest, Ford‐Gilboe, Merritt‐Gray, & Berman, 2003; Wuest, Merritt‐Gray, & Ford‐Gilboe, 2004). Intrusion consists of factors that interfere with women's capacities to manage their health and take control of their lives often at considerable cost to women, families, communities, and health and social systems (Varcoe et al., 2011). Women experience multiple forms of intrusion including unwanted life changes, ongoing abuse, negative health consequences of IPV that persist even after women leave their abusive partners, and personal and financial “costs” of getting help. For example, women with children who face threats to the safety of their children from abusive partners incur financial costs related to supervised access and legal proceedings and often find such services unhelpful (Varcoe & Irwin, 2004; Wuest, Ford‐Gilboe, Merritt‐Gray, & Lemire, 2006). In the grounded theory research, we identified six areas in which women worked to enhance their capacities to limit intrusion; we used those six areas and the sub‐processes within those areas as a basis for the components of *i*HEAL. In our subsequent longitudinal research, we examined these forms of intrusion in detail (Davies et al., 2015; Ford‐Gilboe et al., 2009; Guruge et al., 2012; Ponic et al., 2012; Varcoe et al., 2011; Wuest et al., 2009, 2010, 2008), and we used the findings to inform the development of *i*HEAL.

**Table 1:** Comparison of *i*HEAL and AWI Conceptual Grounding

*i*HEAL Principles	Aboriginal Women's Intervention (AWI) Principles
Safety first:	Being Aboriginal is a strength
The woman's emotional and physical safety will be promoted in all interactions.	The intervention will present connecting to Aboriginal ancestry, history, culture and tradition as a strength; we will seek avenues to proactively counter images and ideologies that pathologize or stigmatize.
Health as priority:	Identity is a priority:
The woman's physical, mental, emotional, and spiritual health will be prioritized.	The extent to which identity erosion has been a form of violence with traumatic effects for Aboriginal people will be recognized and addressed.
Woman‐centered:	History is in present:
The woman will direct the pace, what is given priority within the intervention, and who is involved.	Both traditional culture and colonization will be seen as playing out in the present, rather than being artifacts of the past.
Strengths‐based:	Diversity is valued:
The woman's strengths and capacities will be recognized, drawn upon, and further developed.	The diversity of indigenous people is acknowledged continuously—not only the diversity among Nations, but among individuals who will have had varied histories of connection to their traditions and cultures and to experiences of colonial conquest.
Learn from women:	Woman‐led:
The experiences of the woman and other women who have experienced IPV will be used as a key source of knowledge to help the woman reflect on, talk about, and name her experiences, concerns, and priorities.	The woman choses whether and how to engage in the intervention throughout; interventionists take the lead from women in all aspects
Women in context:	Culture and tradition:
The woman's context of family and close relationships as she defines them will be taken into account.	Both the interventionists and the women will be supported to draw upon tradition and culture to whatever extent they wish—both in terms of collective and individual action.
Calculated risks:	Women in historical and cultural context:
The woman will be supported to assess, judge, and take calculated risks necessary for moving forward.	Attention specifically will be focused on the woman in the context of her history, the history of her family and her nation; women's personal experiences of violence will be connected to these histories.
“Costs” limited:	Healthy interventionist:
The costs of getting help, including from the nurses and the intervention, will be assessed and limited as much as possible.	Emphasis will be placed on supporting the wellbeing of the elders, nurses, and others working as interventionists so that they can foreground the women's agendas.
Support or active system navigation:	Sustainability:
The woman will be helped to seek and obtain support from her community and services, and to deal with the barriers she encounters.	Sustainability of the work beyond the research funding will be woven into the work from the outset, including the training of peer “interventionists.”
Advocacy:	Cultural safety:
The interventionists will work to advocate for improved system responses to women receiving the intervention as they are variously situated within broad social systems of inequity.	Moves practice beyond the notion of cultural sensitivity to more actively address inequitable power relations, discrimination and racism, and the ongoing impacts of historical injustices on health and health care; puts the onus for safety in care on the provider

*i*HEAL was designed to be delivered by a registered nurse (RN) working within the scope of professional practice, in collaboration with an advocate (e.g., a domestic violence worker) over 6 months through approximately 12–14 face‐to‐face meetings in women's homes or safe locations. Ten principles originally guided the intervention (shown in white, Fig. 1), emphasizing collaborative partnerships among the nurse, woman, and other service providers and focusing on health and safety in the context of women's resources, relationships, and community services (Table 1). Using these principles, and a three‐phase process: “Getting In Sync” (1 month to get to know one another), “Working Together” (4 months), and “Moving On” (1 month to phase out the relationship), interventionists work with women to *explore intrusion*, *share options* and *strengthen capacity* within the six components derived from the grounded theory (Ford‐Gilboe et al., 2011):

1. Safeguarding—assessing physical and emotional safety and developing strategies to manage risks and build her sense of security;
2. Managing basics—getting material and economic resources, energy and skills;
3. Managing symptoms—reducing the distress associated with symptoms (e.g., pain, sleeplessness);
4. Renewing self—identifying personal needs, desires, feelings and abilities, and enhancing capacity to make time for herself;
5. Regenerating family—taking stock of family interaction patterns and developing strategies to enhance relating;
6. Cautious connecting—evaluating the costs and optimizing the benefits of current and potential relationships with peers, extended family, social networks, or service agencies.

Nurses support women to identify their priorities both in terms of which areas they wish to work on and their particular priorities within each area. For example, one woman might focus on intrusive symptoms such as chronic pain and insomnia; another experiencing harassment and ongoing abuse might focus on strategies to enhance her safety. The nurses then engaged in supporting the women through listening, teaching, referrals, and practical assistance such as accompanying them to appointments.

The *i*HEAL has been tested in two feasibility studies in different contexts, with significant improvements in health and quality of life (Ford‐Gilboe et al., in review; Wuest et al., 2015). However, the extent to which it would meet the needs of Indigenous women was not known. Our partners at a primary healthcare (PHC) organization serving Indigenous people were seeking to improve services for women. Given the focus on strengthening women's capacities, we anticipated *i*HEAL could provide the basis for an intervention appropriate to Indigenous women's lives and the community. In this study, we aimed to develop an intervention to promote the health of Indigenous women. The purpose of the phase reported here was to develop, pilot test, and refine the intervention.

## Modifying the Intervention for Indigenous Women

Our process to adapt *i*HEAL for Indigenous women in urban contexts was underpinned by critical theoretical and decolonizing approaches (Browne, Smye, & Varcoe, 2005, 2007; Shahjahan, 2005; Stanton, 2014; Zavala, 2013). Importantly, we understood the disproportionate risks of violence for Indigenous women as being created by ongoing colonial relationships between Indigenous people and the Canadian state (Browne, Varcoe, & Fridkin, 2011; Brownridge, 2008; Daoud et al., 2012; Maddison, 2013; Pedersen et al., 2013; Varcoe & Dick, 2007, 2008). This relationship has been characterized by policies that dispossess Indigenous people of their lands, culture, traditions, livelihoods, and children (Dion Stout, 2012; Fast & Collin‐Vezzina, 2010; Firestone et al., 2014; Peters & Anderson, 2013; Royal Commission on Aboriginal Peoples, 1996a). The greatest risk was that building on *i*HEAL, and attempting to “indigenize” an intervention created for, and piloted with, non‐Indigenous people, would impose another colonizing intervention. A second risk was contributing to medicalizing and pathologizing discourses that undermine the area's residents (Liu & Blomley, 2013) and Indigenous women (Tang & Browne, 2008). Our approaches were intended to mitigate these risks.

### Steering Committee of Indigenous Women

Indigenous women leaders from academia, community, and health care with expertise specific to IPV and to the context formed the Steering Committee and guided the study from funding application to present. These women have been team members on previous studies informing the current research.

### Articulating an Indigenous Lens

We articulated an “Indigenous lens,” drawing on literature from Indigenous scholars regarding Indigenous knowledge and approaches and historical trauma. Author MDS, a Cree‐speaker, used Cree concepts to articulate key aspects of the intervention and broaden our thinking. For example, in contrast to the English word “poverty,” which refers primarily to economic deprivation, the Cree word kitimitkasóna implies a plurality of intersecting poverties (Dion Stout, 2012, p. 12), as seen in the study setting in which women experienced multiple challenges to their physical, emotional, mental, and spiritual needs. Cree concepts were presented within three larger conceptual areas: *i*HURT, the areas of intrusion experienced by Indigenous women; *i*HEAL, areas in which healing might be concentrated, and *i*HELP, which attended to women's expressed desire to give back and help others, a theme seen throughout clinical practice and research with women who experience violence (Browne, Smye et al., 2011; Draucker et al., 2011; Valpied, Cini, O'Doherty, Taket, & Hegarty, 2014).

### Interviews With Indigenous Elders

We conducted qualitative interviews with Elders (*n* = 10) from various Nations. The Elders were living in the study setting and knowledgeable about colonial policies and Indigenous women, traditions, culture, and IPV. Elders are acknowledged as revered leaders in many Indigenous communities; their leadership typically arises not from their age but from recognition of their knowledge, experience, and wisdom by the wider community. We sought guidance to make the intervention appropriate and effective, including teachings to support women to improve their health. In analysis of these interviews, we identified that the intervention needed to (a) be inclusive, holistic, and trauma‐informed; (b) foreground the healing power of traditions and culture; (c) incorporate the teachings and wisdom of Elders; (d) make spaces and programs as welcoming and safe as possible; and (e) be offered by interventionists able to understand the women's needs and engage in partnership. A recurring theme was that to address the intertwined effects of structural and interpersonal violence, we needed a holistic intervention that offered unconditional physical, mental, emotional, and spiritual supports. Another theme was that traditional cultural practices might motivate engagement and reinforce and revive positive identities. The Elders stressed a need to incorporate the healing power inherent in traditions such as drumming and singing, and to view such practices as therapeutic interventions. However, they cautioned that not all women would be “at the same place in their healing journey”; given the diversity of women's cultural backgrounds and experiences, they might not have common traditions, and some might not be connected to their cultural heritage. We needed to respect each woman's preferences rather than imposing cultural practices.

## Revision of Intervention

### Revised Principles

Guided by the steering committee, the Indigenous lens, Cree concepts, interview results, and our prior research, we derived 10 new principles (shown in dark shading, Fig. 1) for the Aboriginal Women's Intervention (AWI) study, which complemented and extended the 10 existing *i*HEAL principles. All 20 principles informed the revised intervention. For example, given that colonial policies have resulted in extensive surveillance, regulation (e.g., of property rights), and control over Indigenous women, we shifted from intending to learn from women and be woman‐centered to intending to be woman‐led. Given efforts by the Canadian state to erase Indigenous identity, we purposefully drew attention to how being Aboriginal is a strength, and we focused on identity as a priority.

Our principles included attending to the women's contexts, including their social, economic, cultural, and historical contexts. Thus, given our research on Indigenous women's experiences of intimate partner violence (Varcoe & Dick, 2007, 2008) and “leaving” abusive relationships (Smye et al., 2017), and the known limits to women's options living in the community (Benoit, Carroll, & Chaudhry, 2003; Culhane, 2003; McNeil, Shannon, Shaver, Kerr, & Small, 2014; Miewald & Ostry, 2014; Peters & Anderson, 2013; Robertson & Culhane, 2005), we decided to not restrict eligibility to women who had recently left an abusive partner as we had done in prior feasibility studies.

### Revision of Intervention Materials and Approaches

Working from these principles, we revised the intervention materials, including the theoretical and empirical foundations, sample scripts used to train the nurses, and tools for use with women. Specifically, we (a) reduced class bias by removing assumptions such as that women would have homes or access to child care or a car; (b) attended to the fact that women may not have left abusive partners; (c) integrated greater attention to the diversity of women along multiple lines of difference and identity (e.g., ability, sexual and gender identity, Indigenous identity); and (d) recognizing the extent of state apprehensions of Indigenous children and over‐involvement of the state in child rearing of Indigenous children in Canada (Blackstock, 2007; Blackstock, Trocmé, & Bennett, 2004; McKenzie, Varcoe, Browne & Day, 2016; Sinha, Trocmé, Blackstock, MacLaurin, & Fallon, 2011; Trocmé et al., 2010), eliminated the assumption that women would have their children living with them. We also integrated recommendations from the feasibility studies (Ford‐Gilboe, Varcoe et al., 2017; Wuest et al., 2015) to (a) provide opportunities for women to meet and potentially support each other; (b) integrate more attention to substance use including training for nurses; and (c) pay more attention to spirituality.

Building on advice of the Elders, a steering committee member (author RP) led the development of the role of Elder to work in partnership with the nurses to deliver the intervention, integrating the use of a Circle. The Circle is a common concept among Indigenous peoples; there are many forms of Circles with varying levels of formality and ceremony, such as learning Circles, decision‐making Circles, sharing Circles, and healing Circles. We envisioned the Elder's role to be one of offering teachings about culture and traditional practices and supporting spirituality within an information Circle. We initially invited women to an Elder‐led Circle as an information and recruitment opportunity to meet the nurses and other women and offered the Circle as part of the intervention.

For pilot‐testing, the revised intervention retained all features of the previous versions of *i*HEAL: the nurses worked 1:1 with the women on their priorities within the six components over approximately 6 months. However, ROS was augmented by a Circle attended by the nurses and led by an Elder who used ceremony and taught cultural and traditional practices. Furthermore, following from the newly developed principles, the nurses’ work was informed by training about the history and contemporary circumstances of Indigenous people.

## Pilot Testing

The pilot study was conducted to examine the feasibility of implementing the revised intervention with Indigenous women, assess its acceptability to the women and providers, and gather initial data on key outcomes prior to conducting a larger study. Three RNs and one Elder with extensive experience working in the study setting and with Indigenous women were hired to deliver the intervention and provided with 45 hours of training related to the intervention theory and components and the dynamics and health effects of violence. The Circles, nurse's offices, and meeting spaces were hosted initially by our Primary Health Care (PHC) partner organization. Indigenous women were invited to meet individually with an assigned nurse on a weekly or biweekly basis and to attend the Circles. Many lessons were learned during the pilot study. We initially intended to delay the start of intervention Circles until all women had been recruited and then institute them biweekly. However, as the intervention began, based on their experiences of the recruitment Circles, the women indicated they wanted frequent and regular Circles, and some were initially reluctant to meet 1:1 with the nurses, largely due to prior negative experiences with health care. Therefore, we continued the Circles on a weekly basis, while women engaged in less 1:1 time with nurses than expected. The Circles evolved from an information and recruitment Circle to a cultural teaching Circle to “sharing Circles” in which the Elder and nurses provided emotional, spiritual, and social support to the women. Throughout, the Circles emphasized the importance of cultural protocols and traditional teachings such as an opening prayer, formal introductions, using a talking feather, smudging,^2^ and culturally based activities (such as making medicine pouches).

### Ethical Considerations

We received approval from the Ethics Review Boards of the investigators’ universities. We adhered to national standards regarding conducting research with Indigenous peoples (Canadian Institutes of Health Research, 2010), including training for all staff. Key considerations included avoiding coercion to participate, protecting confidentiality within a group context and limiting emotional harm. In the context of extreme poverty, offering food, bus tickets and honoraria can coerce participation. Consequently, we employed strategies such as providing honoraria immediately following obtaining informed consent prior to collecting data, and providing food without expectation that women would stay for the Circle. We sought women's feedback and monitored and adjusted our practices as the pilot progressed.

### Recruitment and Sample

Twenty‐one adult women who (a) identified as Indigenous; (b) had experienced violence from an intimate partner (but had not necessarily left the partner); (c) spoke and understood English; and (d) lived in the study community were recruited through our partner agency and word‐of‐mouth. Women were invited to learn about the study at an Information Circle led by the Elder. Interested women were given information materials. The Abuse Assessment Screen (AAS; McFarlane, Parker, Soeken, & Bullock, 1992), a brief, well‐established tool shown to reliably confirm the presence of past and present IPV in diverse women (Laughon, Renker, Glass, & Parker, 2008; Wuest et al., 2009, 2008), was used to screen for experience of IPV. Following informed consent, measures were administered by a research assistant trained in data collection with Indigenous women and women who have experienced violence. Women were invited to tell others about the study, and undecided women were invited to attend a second Information Circle. Unintentionally, the information and recruitment Circle drew regular attendance; women returned even after they had joined the study and completed their pre‐intervention surveys, indicating that they “wanted more” of the Circle. These potentially confounding dynamics underscore the importance of pilot testing.

### Data Collection

We collected pre‐ and post‐intervention data using standardized instruments to assess demographics, abuse experiences, health and service use, mental health, chronic pain, quality of life, agency, mastery, social support, and conflict. Pre‐intervention data collection time averaged 45 minutes to 2.5 hours (average 1.5). Post‐intervention data collection time averaged 2 hours and included qualitative interviews focused on individual experiences of the intervention.

Twenty‐one women completed pre‐intervention surveys; 12 women completed both pre‐ and post‐intervention surveys. Sixteen women participated in individual post‐intervention interviews (four had not completed surveys), including those who participated in all aspects, only in Circles, only met with nurses 1:1, or withdrew immediately after initial recruitment. Thirteen women who completed individual interviews additionally participated in a focus group to solicit group feedback. We complemented these data with interviews of the nurses (*n* = 3) and Elders.

Standardized and investigator‐developed self‐report measures selected in line with our grounded theory were used to create a descriptive profile of the women's demographic characteristics, abuse experiences, current health problems, and health and social service use, and measure changes in key outcomes pre‐ and post‐intervention.

### Descriptive Measures

#### Demographic measures

Demographic questions included consideration of socio‐economic conditions (employment status, income, and work experiences), presence of dependent children, receipt of child support, housing situation, and shelter use in the past year. We also collected data on cultural background, languages spoken, and self‐identification with Indigenous communities (i.e., status First Nations, non‐status First Nations, Inuit, and Métis). The Financial Strain Index (FSI; Ali & Avison, 1997) was used to assess women's levels of difficulty managing financial obligations (Table 2).

**Table 2:** Descriptive Measures

	Variable	Measure	Items	Description. Participants Were Asked:
Demographics	Financial strain	Financial strain index (FSI)^a^	12	To rate their difficulty meeting financial obligations on a 4‐point scale ranging from very difficult (1) to not at all difficult (4).
Abuse experiences	IPV experiences	Modified abuse assessment screen (AAS)^b^	4	If, in the past 12 months, they had experienced 4 types of IPV from a partner (physical abuse, forced sex, fear of partner, or coercive control) and, if so, the number of times.
	Child abuse experiences	Modified questions from Childhood Trauma Questionnaire (CTQ)^c^	4	Whether they believed they had experienced physical, emotional/mental, sexual, and/or spiritual abuse as children on a 5‐point scale ranging from never true (0) to very true (4).
	Sexual assault	Violence against women survey^d^	2	Whether or not they have experienced sexual assault since the age of 16 as defined by the Canadian Criminal Code. They were asked if they had been (a) touched against their will in a sexual way and (b) forced into sexual activity.
Health and service use	Symptoms/health problem	Partner abuse symptom scale (PASS)^e^	45	Whether they had experienced each of 45 symptoms in the last 12 months (yes/no)
	Self‐rated health	Short‐form general health survey version 2 (SF‐12v2)^f^	12	To rate their health from excellent (1) to poor (5) based on perceived health over the past four weeks. Raw scores are transformed to 0–100 scale score, higher scores reflect greater health functioning.
	Health and social services	Author‐developed measure^g^	27	Whether they had used specific health and social services in the past month and to rate how the services met their needs, from not well (1) to very well (4). Participants were asked about various services including doctors, counsellors, crisis phone lines, and food banks.

^a^ Ali and Avison (1997).

^b^ Parker and McFarlane (1991).

^c^ Bernstein and Fink (1998).

^d^ Johnson and Sacco (1995).

^e^ Ford‐Gilboe, Campbell et al. (2017).

^f^ Ware et al. (1996); Ware, Kosinski, Turner‐Browker, and Gandek (2002).

^g^ Ford‐Gilboe et al. (2015).

#### Abuse experiences

Collection of women's abuse histories was limited to minimize potential harm. In addition to the AAS to assess IPV, women were asked about sexual assault and harassment using two items from the Canadian Violence Against Women Survey (Johnson & Sacco, 1995). Exposure to physical, sexual, and emotional child abuse was assessed using questions from the Childhood Trauma Questionnaire (Bernstein & Fink, 1998); women found these questions triggered difficult memories and were very distressing to complete.

#### Health and service use

Information about current health problems was gathered using self‐report responses (yes/no) to 45 health problems associated with IPV using the Partner Abuse Symptom Scale (PASS), an investigator‐developed tool (Ford‐Gilboe, Campbell et al., 2017) used previously with women who have experienced IPV (Wuest et al., 2008). Self‐rated health was measured using the short‐form General Health Survey Version 2 (Ware, Kosinski, & Keller, 1996). Regarding health and social service use, women were asked whether they had used specific services in the last month and, if used, to rate how the service fit with their needs. These questions have been used previously with women experiencing IPV (Ford‐Gilboe et al., 2015).

### Outcomes Measures

Seven self‐report scales were used to measure pre‐post intervention changes in key outcomes (Table 3). Higher scores on all scales reflect higher levels of the variables being measured.

**Table 3:** Summary of Pre‐ and Post‐Intervention Measures

Variable	Measure	Items	Description and Scoring	Reliability
Depression	CESD‐R^a^	20	Symptoms of depression based on DSM criteria, rated on a 5‐point Likert scale. Participants are asked to report how often they have experienced these symptoms in the past week or so from Not at all or less than 1 Day (0) to Nearly every day for 2 weeks (4). The highest scores for each item are converted from 4 to 3 and items are summed with a possible range of 0–60, to make scores consistent with the original CESD scores. Cut‐off score of 16 is an indicator of possible clinical depressive symptoms.	alpha:
				.93 (this study)
				.90–.96^b^
Symptoms of trauma	PCL^c^,^d^	17	Based on DSM‐IV criteria for PTSD. Participants report how much each symptom has bothered them in the past month from Not at all (1) to Extremely (5). Items are summed with a possible range of 17–85. A cut‐off of 44 is used as indicator of probable clinical trauma symptoms.^c^	alpha:
				.86 (this study)
				.94^c^
Chronic pain	Von Korff pain grade scale^e^	7	3 items measuring pain intensity each scored on a scale from 0–10. 4 items measuring pain disability, 3 items scored on a scale from 0–10, and 1 item reporting number of disability days in the past 6 months. These scores are transformed to generate: pain intensity score (0–100), disability score (0–100), and pain grade classification (Grades 0–4):	alpha:
				.91^f^
				Pain intensity
				.78 (this study)
				Pain disability
				.84 (this study)
			• 0: Pain free	
			• I: Low disability low intensity	
			• II: Low disability high intensity	
			• III: High disability moderately limiting	
			• IV: High disability severely limiting	
Quality of life	Sullivan quality of life scale^g^	9	Adapted from a longer measure of quality of life (well‐being across key life domains) developed by Andrews and Withey (1976) to capture the quality of life among women with histories of IPV. Participants are asked to rate each item on a 7‐point scale measuring satisfaction with particular areas of their lives from extremely pleased (1) to terrible (7). Responses are reverse‐scored and summed with higher scores indicative of higher quality of life, scores range from 9 to 63.	alpha:
				.88 (this study)
				.85–.88^h^
Agency	Personal agency and interpersonal agency scales^i^,^j^	8, 5	In the personal agency scale participants report how often they use their own efforts, abilities and skills in order to accomplish their goals from never (1) to often (4). Responses are summed and with total scores of 8–32. In the interpersonal agency scale, participants report how often they work with other people in order to achieve their goals from never (1) to often (4). Responses are summed with total scores of 5–20.	alpha:
				Personal agency
				.90 (this study)
				.78^j^
				Interpersonal agency
				.88 (this study)
				.76^j^
Mastery	Pearlin mastery scale^k^	7	Rate how much they agree/disagree they feel in control over their life circumstances from strongly agree (1) to strongly disagree (5). Scores are transformed so that higher scores indicate higher perceived mastery (5–35).	alpha:
				.82 (this study)
				.65–.88^l^,^m^,^n^,^o^,^p^,^q^,^r^
				Test–retest:
				.44–.66^p^,^s^,^t^
Social support and social conflict	Interpersonal relationships inventory: social support and social conflict^u^	13,13	Social support: How much they agree/disagree that they have helpful social support from strongly disagree (1) to strongly agree (5), or how often they have helpful social support from never (1) to very often (5).	Social support
				alpha:
				.81 (this study)
			Social conflict: how much they agree/disagree that they have social conflict or stress in their relationships from strongly disagree (1) to strongly agree (5), or how often they have social conflict or stress in relationships from never (1) to very often (5). Subscales are summed separately with scores ranging from 13–65. Higher scores represent higher levels of social support and social conflict.	.92^u^
				.79–.95^v^
				Test–retest:
				.91^u^
				Social conflict
				alpha:
				.86 (this study)
				.91^u^
				.68–.92^v^
				Test–retest
				.81^u^

^a^ Comstock and Helsing (1976); Eaton, Smith, Ybarra, Muntaner, and Tien (2004); Radloff (1977).

^b^ Van Dam and Earleywine (2011).

^c^ Blanchard et al. (1996).

^d^ Weathers, Huska, and Keane (1991).

^e^ Von Korff (1992).

^f^ Smith et al. (1997).

^g^ Sullivan, Bybee, and Allen (2002).

^h^ Bybee and Sullivan (2005); Goodkind, Gillium, Bybee, and Sullivan (2003); Gillium, Sullivan, and Bybee (2006); Sutherland, Bybee, and Sullivan (2002); Sullivan et al. (2002).

^i^ Smith et al. (1999).

^j^ Smith et al. (2000).

^k^ Pearlin and Radabaugh (1976); Pearlin and Schooler (1978).

^l^ Greenberger and Litwin (2003).

^m^ Hobfoll, Schröder, Wells, and Malek (2002).

^n^ Marshall and Lang (1990).

^o^ Morisky et al. (2001).

^p^ Nolen‐Hoeksema, Larson, and Grayson (1999).

^q^ Schieman and Turner (1998).

^r^ Turner, Pearlin, and Mullan (1998).

^s^ Brady (2003).

^t^ Pearlin, Menaghan, Lieberman, and Mullan (1981).

^u^ Tilden, Nelson, and May (1990).

^v^ Tilden, Hirsch, and Nelson (1994).

#### Mental health

Two widely used mental health measures were used: the 20‐item Center for Epidemiological Studies Depression Scale‐Revised (CESD‐R; Eaton, Smith, Ybarra, Mutaner, & Tien, 2004), and the Post‐Traumatic Stress Disorder Checklist for civilians (PCL‐C), a measure of the severity of trauma symptoms (Blanchard, Jones‐Alexander, Buckley, & Forneris, 1996).

#### Chronic pain

The Chronic Pain Grade Scale was used to capture the multi‐dimensional nature of chronic pain (intensity, persistence, and pain‐related disability) in a graded classification (Von Korff, 1992). While the reliability and validity of the Chronic Pain Grade scale has been demonstrated in community and clinical samples of adults (Mallen, Peat, Thoma & Croft, 2005; Plesh, Crawford & Gansky, 2002), internal consistency of the pain intensity score in this study was lower than expected (Cronbach alpha = .78).

#### Quality of life

Sullivan's Quality of Life Scale (Sullivan & Bybee, 1999), a brief 9‐item measure developed for and validated with women who have experienced IPV (Bybee & Sullivan, 2002, 2005) was used. Items for this scale were adapted from those developed by Andrews and Dilthey (1976) in a classic study of psychological well‐being among US population samples.

#### Agency, mastery, social support, and conflict

Women's sense of agency (“one's own efforts and abilities to achieve desired consequences”) was measured using the Personal Agency Scale (PAS) and Interpersonal Agency Scale (IAS; Smith et al., 2000, p. 463). The woman's sense of control over her life was measured using the Pearlin Mastery Scale (Pearlin & Radabaugh, 1976). The Interpersonal Relationships Inventory (Tilden, Nelson, & May, 1990) was used to measure social support and social conflict.

The women's intervention participation profiles (Circle attendance, meeting number, duration) and health information (health and medical histories, blood pressure, medications, referrals) were recorded by nurses in structured participant files. Nurses’ and elders’ hours of work, kilometers traveled, and costs were recorded.

### Data Analysis

Descriptive statistics were used to summarize study variables. Pre‐ and post‐intervention measures were compared but not tested statistically due to the small sample size. We conducted an interpretive thematic analysis of individual and group interviews data using procedures for qualitatively derived data (Braun & Clarke, 2006; Denzin & Lincoln, 2011; Thorne, 2008), using NVivo™ to assist with organizing and coding the interviews. Interview transcripts were read repeatedly to ascertain recurring and contradictory patterns in the data and possible linkages to theoretical perspectives. As data were collected and analyzed, the analysis shifted to a more conceptual representation. As suggested by Carey and Smith (1994), we analyzed the focus group data to consider individual perspectives, interactions between individuals, and the group perspective. Finally, we compared individual and focus group data. There were no contradictions between individual and focus group interviews, and in the focus group women reached consensus on their key suggestions for improving the intervention.

## Pilot Study Findings

### Sample Description

The 21 participants identified as being from a wide range of Indigenous Nations and communities. All lived with considerable financial insecurity, with the majority unemployed and on disability assistance (Table 4). Over half lived in unstable or unsafe housing (e.g., single room occupancy hotels, shelters). From clinical histories and self‐report, it was clear that all women had experienced extensive IPV and racism; most had experienced sexual assault and child abuse.

**Table 4:** Characteristics of Participants in Pilot Study

Characteristic	*n*	Range	*n*	%	*M*	*SD*
Age in years	21	32–58			46.2	7.6
Employed full or part‐time	21		6	28.5		
Unemployed	21		15	71.4		
On disability assistance	21		18	85.7		
Spent one or more nights at a shelter during the past 12 months	21		9	42.9		
Permanent change in residence during the past 12 months	20		8	40.0		
Difficulties paying for housing	21		19	90.5		
Experienced sexual assault (since 16 years old)	19		14	73.7		
Experienced abuse from a partner during the past 12 months	21		8	38.1		
Experienced abuse as a child	19		17	89.5		

*Note*. *M*, mean; *SD*, standard deviation.

### Intervention Uptake

Participation varied greatly and was inconsistent for some women. Consequently, the Circle size varied from week to week (3 to 15), and women missed many appointments with the nurses. The nurses expended considerable effort reminding the women of appointments and the Circle schedule and assisting them with transportation to get to the Circle. Many women experienced memory problems, and the realities of poverty, traumatic life events (such as deaths of friends and family), and ongoing violence created significant challenges for planning and following through.

A total of 28 Circles were held over 6 months. Nine women (43%) attended half or more Circles. Post‐test data were not collected from three women who did not engage with the nurses in any 1:1 visits; the remaining 18 women spent an average total contact time, including Circles and 1:1 nurse visits, of 28.2 hours; of that, an average of 6.25 hours was spent with the nurses (range 1–14.5). This was more overall time but less 1:1 time with nurses than in the feasibility studies, in which women averaged 16.8 and 15.9 hours of contact with interventionists (Ford‐Gilboe, Varcoe et al., 2017; Wuest et al., 2015).

### Health and Service Use

Over 90% of the women reported at least one active health condition. Consistent with the demographic profile of the study site, 11 women (52%) reported being HIV‐positive, 8 reported hepatitis C virus infection (HCV). The majority reported prior head injuries and identified as currently having a substance use problem. Symptoms affecting over half of the women included: difficulty sleeping, fatigue, feeling sad or depressed, back pain, swollen and painful joints, headaches, difficulty concentrating, upset stomach/heartburn, general aches and pains, and bowel problems. All but one woman were living with chronic pain: 13 (62%) with mild to moderate chronic pain, and 7 (33%) with highly disabling chronic pain (i.e., grade 3 or 4 on the Chronic Pain Grade Scale). Despite this, the women rated their overall health as fair (38.1%), good (38.1%), or very good (23.8%). The women were well‐connected to services; 17 had accessed counseling in the prior month, and 16 of the 18 who answered reported seeing a family doctor in the past month, which 14 of 16 rated as fitting “well” or “very well” with their needs.

### Pre‐Post Changes in Outcomes

Twelve women provided pre‐ and post‐intervention data. The overall participation of these women ranged from 20 to 58.5 hours (*M* = 40.2, *SD* = 11.99); they spent an average of 7.7 hours with nurses in 1:1 meetings. Although the sample size was too small for statistical testing, a pre‐post trend was observed of decreased depressive and trauma symptoms, and increased sense of control over their lives (personal and interpersonal agency) and QOL (Table 5). Ten women met the threshold for probable depressive symptoms pre‐intervention, and eight women post‐intervention, using a cut score of 16 on the CESD‐R. Pre‐intervention, eight women had significant trauma symptoms, in comparison to six women post‐intervention, based on a cut score of 44 on the PCL‐C.

**Table 5:** Comparison of Trends in Selected Pre‐ and Post‐Intervention Scores (*n* = 12)

		Pre‐Intervention		Post‐Intervention		
Variable	Range	*M*	*SD*	*M*	*SD*	Direction of Difference
Depressive symptoms	0–56	28.8	13.6	23.4	16.9	Lower depressive symptoms
Trauma symptoms	20–75	50.6	11.0	41.4	15.1	Lower PTSD symptoms
Interpersonal agency	5–20	13.3	4.4	15.3	4.1	Greater interpersonal agency
Personal agency	14–32	24.4	6.6	27.6	4.1	Greater personal agency
Quality of life^a^	16–63	40.0	9.9	44.1	12.6	Improved quality of life
Social support	34–64	52.4	7.6	52.2	8.5	No change
Social conflict	19–57	44.6	7.0	41.1	9.2	Lower social conflict
Mastery	15–35	24.3	6.0	25.0	6.0	No change
Pain disability	0.0–100	38.3	22.5	52.8	24.6	Greater pain disability
Pain intensity	33.3–90.0	64.4	18.6	65.8	16.4	Greater pain intensity

*Note*. *M*, mean; *SD*, standard deviation. Trends are descriptive of differences in average scores and not based on statistical testing due to small *n*. Mean replacement, using participants’ mean item scores, was used for participants with one missing item in a particular measure.

^a^ One participant who completed the pre‐ and post‐intervention surveys did not answer the questions in this scale.

### Intervention Acceptability

Based on individual and focus group interviews, women found the intervention acceptable overall and found some aspects especially helpful: checking in during the Circle to share how they were feeling, prayer and traditional teachings, smudging, the talking feather, traditional activities, food during the Circles, and access to nurses. Women noted that participating improved their confidence, openness, trust with others, and outlook. They reported a greater sense of connection to others, greater safety, increased hope, and saw the intervention as an important “step on the healing journey.” They reported seeing themselves not only as survivors of their experiences but as fighters striving to improve their own lives and those of all Indigenous women.

Although satisfied with the intervention overall, the women identified challenges that deterred participation. Given their high levels of loss, trauma, and grief, it was not surprising that being triggered^3^ was the most commonly cited challenge. Women described being triggered by stories of trauma and loss, which were profoundly upsetting to some, causing them to leave the Circle or “tune out.” While staff encouraged women not to use substances when attending the Circle, some women (especially those trying to reduce their own use) were triggered when others discussed substance use. The nurses and Elder worked to limit these occurrences and support women when distressed, but the women thought staff needed more training and skill.

The women found conflict between group members and among study staff challenging. In fact, the level of conflict necessitated moving the meetings to a community center from the PHC partner organization where some women were receiving health care and where some intervention staff were employed. Some women thought certain participants were allowed to dominate; some women were not interested in “cultural” approaches, with one woman saying she would like to “get to the root of the problem rather than playing with beads.” Some women and staff worried that some women participated only for food and the option of bus tickets or $5, but they also understood such incentives were supportive to women in extreme poverty. One woman highlighted complex group dynamics, saying, “Most of them are addicts. They're there to get their five bucks to go get their next toke or their next drink, whatever the case may be. However, at the same time, they're there.”

In interviews, the women offered important recommendations. Most wanted smaller Circles to facilitate more opportunity for each women to speak and to limit the number of difficult stories being told. Women wanted more traditional teachings, trauma counseling, information on IPV and safety planning, and to have the Circle led by more than one Elder, especially to integrate more diverse traditions. All agreed more structure was needed for the Circles and strongly recommended hiring nurses with better understanding of Indigenous women's lives. Most wanted more opportunity to work 1:1 with the nurses; it was disappointing to learn that 6 of 13 women in the final focus group did not know they could access the nurses outside of the Circle. On review, we identified that although information had been provided, we could have done more to facilitate access, given the women's lives and memory challenges.

## Pilot Study Lessons and Application to Later Efficacy Testing

The pilot was invaluable for revising the intervention to enable the efficacy testing being carried out at present. The pilot helped us to (a) gain a deeper appreciation for the complexity of women's lives and health issues; (b) develop more advanced training for interventionists (c) consider how to support meaningful participation while limiting triggering; and (d) support more effective relationships among intervention staff.

As expected, the women's demographic profiles indicated extreme poverty and high levels of disability and unemployment, and few women lived with their dependent children. Overall, the women's health profiles aligned with what is known about the health of women who experience violence (Campbell & Lewandowski, 1997; Gilchrist, Hegarty, Chondros, Herrman, & Gunn, 2010; Hill, Schroeder, Bradley, Kaplan, & Angel, 2009; Stockman, Hayashi, & Campbell, 2015; Svavarsdottir & Orlygsdottir, 2009; Wuest et al., 2015) and are consistent with our previous research with women who had left abusive partners (Ford‐Gilboe et al., 2009). However, distinguishing characteristics of the pilot sample included the high proportion living with HIV and HCV, reporting head injuries and memory problems, and identifying as struggling with substance use. These findings are understandable given the study context and its concentration of services to address these health issues.

Differences in the pre‐ and post‐measures suggest the intervention may facilitate a decrease in women's depressive and trauma symptoms and increase women's sense of agency, control, and QOL. However, the trend toward worsening pain suggests that the intervention did not help women manage their pain and perhaps increased their awareness of pain. Implications for efficacy testing included (a) further training for nurses regarding HIV, HCV, substance use and chronic pain; (b) intensified support to help women remember and keep appointments; (c) intensified support for women's access to social determinants of health, such as housing and income (d) expanding settings to recruit women with greater financial and social resources and who were directly parenting dependent children.

Women's participation with the nurses was lower than in previous feasibility studies (Ford‐Gilboe, Varcoe et al., 2017; Wuest et al., 2015) but was offset by higher Circle participation, where both an Elder and nurses were present. Women were generally positive about the nurses but unclear regarding the access they had to them and whether Circle attendance was mandatory (it was not, but some thought they would be penalized for not attending). Hired part‐time, the nurses had limited availability. Throughout, the nurses struggled to use the intervention language with the women. For the efficacy study, we determined to prioritize facilitating 1:1 engagement with nurses by communicating the nurse's availability, hiring nurses full‐time, and offering intervention components initially in a workshop format to increase information sharing, decrease potentially traumatizing stories, and allow nurses to support one another when explaining the components.

The Circles with the Elder were an innovation unique to ROS. The women were positive and appreciative of both; many saw the Circle as “the program,” with the nurses as peripheral to their experience. However, some women's participation in the Circle was deterred by “being triggered,” the dynamics among staff, and varied degree of interest in “cultural” approaches. The Elder expected to offer cultural and traditional teachings but was put in a position of leading Circles in which women wanted to share their difficult histories, without support from nurses or others trained to safely facilitate such groups. Thus, staff recommendations for more structure aligned with those of the women. For the subsequent efficacy study, we decided to hire more than one Elder, clarify the role of Elder to minimize negative dynamics arising from role conflict, and use a shared leadership model between nurses and Elders. We decided to increase training for interventionists regarding group facilitation and trauma‐ and violence‐informed care and to hire nurses with community health experience and stronger understanding of Indigenous women's lives (preferably Indigenous nurses).

## Testing Feasibility and Efficacy of Reclaiming Our Spirits

Based on the pilot findings, a refined version of ROS is currently undergoing efficacy testing. We are using a quasi‐experimental, two‐group, time‐series design with a convenience sample of 130 women recruited in a similar manner to the pilot study. Study settings now include a more suburban setting. As in the pilot, the intervention is being delivered by RNs and Elders hired for the study, supported by the research team and other experts. Outcome variables will be measured at baseline and 6 and 12 months later, with some modifications of the instruments based on the pilot experience. In keeping with recommendations for evaluating complex interventions (Egan, Bambra, Petticrew, & Whitehead, 2009; Lewin, Glenton, & Oxman, 2009), we are collecting qualitative and quantitative data from participants and interventionists and conducting an audit of study records to assess fidelity, gain understanding of the process by which the intervention produces its effects, and identify unanticipated outcomes and barriers to implementation.

## Conclusion

The *i*HEAL, including the version developed for Indigenous women described here, is a promising intervention. The pilot results suggest acceptability and promise for Indigenous women. Efficacy testing will build knowledge specific to promoting the health of Indigenous women and other women experiencing IPV.

The Truth and Reconciliation Commission of Canada (2015), which focused on the role of Indian Residential Schools in the cultural genocide effected by the Canadian state against Indigenous peoples, called for all Canadians to contribute to reconciliation. Many women participating in this research are survivors of the residential school system, and all were affected by that system and the wider network of colonial policies through their families and communities, making this research highly relevant to the Commission's call. Those within the healthcare system can contribute to reconciliation and healing for all by understanding how history lives on, including through the strength and resilience of Indigenous women, and by taking that understanding into the provision of care.

## Conflicts of Interest

The authors declare no conflicts of interest.

## Acknowledgments

The authors gratefully acknowledge the contributions of pilot nurses: Doreen Littlejohn, Susan Giles, and Evanna Brennan. We also acknowledge the guidance and expertise of Dr. Judy Wuest who co‐led much of the research leading to the theoretical foundations of the intervention, and the two previous feasibility studies. Most importantly we acknowledge the contributions of the women who participated in the pilot study who provided extensive input formally and informally, and continue to do so. This study was funded by the Canadian Institutes for Health Research Institute for Aboriginal Peoples Health, Operating Grant #231515.

## Endnotes

1 Drawing on the United Nations Declaration on the rights of Indigenous People and the Report of the Royal Commission on Aboriginal Peoples, we use the terms Indigenous people and Aboriginal people interchangeably to refer to the original inhabitants of what is now known as Canada, which includes First Nations, Métis and Inuit people. Our Indigenous partners are mixed in their opinions, but overall our team prefers the term *Indigenous*. However, we use the term *Aboriginal* in places because (a) the research was originally named “the Aboriginal Women's Intervention study”; (b) our participants use the term Aboriginal; and (c) the term may be more familiar.

2 Smudging is a practice common to many Indigenous groups in North America, involving ceremony and often the use of the smoke of sacred herbs, grasses, or tree branches to cleanse one's self and thoughts. Some traditions use elements representing; earth, water air, and fire.

3 “Triggered” was very common parlance in the study setting and was used to refer to being upset, sometimes to the point of having “flashbacks” or feeling as though a traumatizing experience were reoccurring.
